# Absolute lymphocyte count trajectory predicts clinical outcome in severely injured patients

**DOI:** 10.1007/s00068-025-02864-0

**Published:** 2025-05-02

**Authors:** Lena-Marie Reichardt, Bianca Hindelang, Lönna Süberkrüb, Kim Lena Hamberger, Jan A. Graw, Konrad Schuetze, Elisabeth Zechendorf, Marco Mannes, Rebecca Halbgebauer, Lisa Wohlgemuth, Florian Gebhard, Markus Huber-Lang, Borna Relja, Christian B. Bergmann

**Affiliations:** 1 Translational and Experimental Trauma Research, Department of Trauma, Hand, Plastic and Reconstructive Surgery, University Hospital Ulm, Ulm, Germany; 2 Department of Anesthesiology and Intensive Care Medicine, University Hospital Ulm, Ulm, Germany; 3 Department of Trauma, Hand, Plastic and Reconstructive Surgery, University Hospital Ulm, Ulm, Germany; 4 Department of Intensive Care and Intermediate Care, University Hospital of the Rheinisch-Westfälische Technische Hochschule Aachen, Aachen, Germany; 5 Institute of Clinical and Experimental Trauma Immunology, University Hospital Ulm, Ulm, Germany

**Keywords:** Lymphopenia, Immunosuppression, Lymphocyte count, Severely injured patients, Immunomonitoring

## Abstract

**Purpose:**

Lymphopenia is associated with adverse clinical outcome in trauma, but no immunomonitoring method is established to identify patients at risk. Absolute lymphocyte count (ALC) represents a promising biomarker and may support clinical decision-making in the intensive care unit (ICU). This study examined the temporal patterns of ALC in severely injured patients and their correlation with clinical outcomes.

**Methods:**

38 severely injured patients with an Injury Severity Score (ISS) of 18 and greater were enrolled. Blood samples were collected on admission and after 8, 24 and 48 h and 5 and 10 days. 38 healthy volunteers served as controls. Patients were classified into four groups after 48 h based on their dynamic ALC: persistent lymphopenia (PL), rapidly decreasing (RD), slowly rising (SR) and normal fluctuation (NF). The groups were compared regarding physical performative outcome - defined as unfavorable when patients died or new functional disability necessitated long term care, in-hospital mortality, ICU length of stay (LOS), and incidence of multi-organ dysfunction syndrome (MODS).

**Results:**

A significant reduction in ALC was observed in all patients over 10 days when compared to healthy volunteers, with all patients trending towards a recovery of their ALC after 10 days. PL and RD were associated with an unfavorable physical performative outcome, increased in-hospital mortality, ICU LOS and incidence of MODS.

**Conclusion:**

The dynamic course of ALC represents a cheap and clinically implementable approach for immunomonitoring within 48 h in severely injured patients. The ALC dynamic may early identify severely injured patients at risk, thus facilitating more informed clinical decision-making.

**Supplementary Information:**

The online version contains supplementary material available at 10.1007/s00068-025-02864-0.

## Introduction

Globally trauma has a significant impact on adolescents and young adults. Recently a worldwide examination found three injury causes in adolescents aged 10–24 years among the top five causes for disability-adjusted live-years (DALY) [1]. Trauma is a leading cause of death and disability, particularly among younger individuals, with more than half of cases resulting in fatality [1]. Furthermore, it represents a significant burden of healthcare system and the economy [1].

Trauma is known to frequently result in lymphopenia [2]. Patients suffering from an higher Injury Severity Score (ISS) are likely more susceptible to lymphocyte apoptosis potentially driving lymphopenia [3]. A severe reduction in absolute lymphocyte count (ALC) significantly increases the risk of infection and infection-related death in the general population [4]. Surgical patients who developed organ dysfunction and, consequently, required intensive care treatment, exhibited a 3.5 times higher mortality rate in cases of persistent lymphopenia [5]. The duration and extent of lymphopenia resulting from lymphocyte apoptosis is suggested to be associated with an elevated risk of infection and mortality in patients with sepsis and other severe injuries such as trauma or burns[6].

Recent evidence highlights the association between lymphopenia and adverse outcomes in patients with multiple trauma [7, 8, 9], 10. These outcomes include an increased risk of secondary infectious complications such as multi-organ dysfunction syndrome (MODS), increased mortality, prolonged hospital stay [7, 8]. Importantly, infectious complications are a major contributor to increased patient mortality, particularly when patients develop sepsis [9]. In addition, a progressive decline in lymphocyte levels is associated with poorer prognosis in trauma patients [8, 10].

Most studies investigating the correlation of ALC and clinical outcome did not consider dynamic changes over time or clustered the patient groups depending on dynamic changes of their ALC over time. To address this gap, Pei et al.. employed an approach where they monitored ALC over time and categorized critically ill patients admitted to a general intensive care unit (ICU) into four groups based on the pattern of the dynamic changes of their ALC over a five day period [11]. The four groups were defined as patients with persistent lymphopenia (PL) a rapidly decreasing (RD) ALC, a slowly rising (SR) ALC and patients with normal fluctuations (NF) of their ALC within normal ranges. Patients with PL or RD ALCs revealed increased mortality rates and were more likely to develop persistent inflammation, immunosuppression and catabolism syndrome (PICS) [11].

Given that this study encompassed a heterogeneous ICU population with a range of diverse underlying conditions, it is pertinent to ascertain whether the identified four lymphocyte progression groups are also applicable to a severely injured patient cohort. Moreover, it would be beneficial for potential clinical evaluation if the categorization could happen earlier than after 5 days.

The present study was designed to test the hypothesis that a rapid decrease in lymphocyte count or persistent lymphopenia is associated with an adverse physical performative outcome, defined as discharge to another hospital, death during hospital stay or necessity of long or short-term nursery, an increased risk of in-hospital mortality, a higher risk of developing MODS and a prolonged ICU length of stay (LOS) compared to trauma patients with normal lymphocyte fluctuations or slowly rising lymphocyte counts. This would probably allow us to use dynamic ALC measuring as a tool to predict outcome in severely injured patients.

## Materials and methods

### Study design

This prospective, single-center, observational study was conducted at the University Hospital Ulm level I trauma center in Germany. The study was approved by the local ethics committee prior to its commencement (No. 65/20 and 260/22 Ethics Committee of Ulm University). Severely injured patients aged 18 years or older with an ISS of 18 or greater were prospectively enrolled between May 2022 and August 2024 in this study (*n* = 38). Patients were excluded if on admission they were anticipated to die within 24 h after admission, underwent preclinical cardiopulmonary resuscitation (CPR), were pregnant, received radiotherapy or chemotherapy in the last 3 months, received immunosuppressive treatment, or were diagnosed with Human Immunodeficiency Virus (HIV) or Tuberculosis (Tbc) at the time of admission. Diagnosis leading to exclusion were ruled out via medical history and if in doubt tested. The clinical information obtained included age, sex and admission parameters of the patients.

Primary clinical outcome was defined as physical performative outcome, in-hospital mortality, ICU LOS and MODS. A favorable physical performative outcome was defined as discharge home either with or without ambulant nursing care. An adverse physical performative outcome was defined as discharge to a nursing home, acute or long-term care facility, a different hospital, or intra-hospital death. A favorable ICU LOS was considered less than 7 days, while adverse LOS was 7 days or more. MODS was defined as a sequential organ failure assessment (SOFA) score of six or greater on at least two consecutive days 48-hours post-admission. Healthy age and sex-matched subjects (*n* = 38) served as controls.

## Blood sample collection

Whole blood was collected upon arrival at the emergency room (0-hour collection timepoint) and at subsequent intervals at 8 h, 24 h, 48 h, 5 days and 10 days post-admission. Blood samples were collected in nine milliliter blood monovettes containing ethylenediaminetetraacetic acid (EDTA) (Sarstedt AG, Nuembrecht, Germany) at the aforementioned timepoints. Samples were collected via either peripheral venipuncture or a central venous or arterial catheter. To ensure optimal sample integrity, the monovettes were maintained on ice during transport to the laboratory, and the blood was then immediately processed further.

### Whole blood count

Complete blood counts were performed by the Clinical Central Facility for Clinical Chemistry. As these are clinical data obtained during the normal course of patient care, the lymphocyte ranges are those established by the local clinical laboratory at the University Hospital of Ulm, Germany. Lymphopenia was defined as an ALC of less than 1.1 × 10⁹ lymphocytes per liter of whole blood, in accordance with the criteria set forth by Drewry et al.. and Warny et al.. [4, 9]. Severe lymphopenia was defined as an ALC of less than or equal to 0.7 × 10⁹ lymphocytes per liter of whole blood according to Drewry et al. [9]. In accordance with the methodology proposed by Pei et al.. [11], patients were divided into four groups based on their dynamic course of systemic lymphocyte concentration over a 48-hours period, whereby a 10% variance was accepted. The approach taken by Pei et al.., required ALC observation over five days following admission to the emergency department [11]. In order to provide an earlier categorization potentially allowing earlier clinical intervention we reduced the time until categorization to 48 h. PL was deemed to be severe lymphopenia for a minimum of 48 h following the traumatic event. RD ALC was defined as an initially normal lymphocyte count of $\:\ge\:$ 1.1 × 10⁹ lymphocytes per liter of whole blood that decreased rapidly to lymphopenia, latest at 48 h post-trauma. SR ALC indicates an initial lymphopenia, defined as an ALC < 1.1 × 10⁹ lymphocytes per liter of whole blood, that gradually increases to normal levels of $\:\ge\:$ 1.1 × 10⁹ lymphocytes per liter of whole blood within 48 h following the trauma. NF was defined as the absence of lymphopenia.

### Statistical analysis

This study compared the admission parameters and different outcome endpoints between the identified trajectory groups, tested negative for normality. For these comparisons, continuous nominal variables were subjected to Mann-Whitney testing for two-group comparisons and Kruskal-Wallis testing for comparisons of more than two groups. Categorical variables were evaluated using Fisher’s exact test with Freeman-Halton extension. The correlation between age and ALC in whole blood was tested by Spearman correlation test. The significance threshold was *p* < 0.05 for all tests performed. Insufficient sample volume or patient unavailability due to clinical intervention rarely accounts for differences in numbers for some parameters. Data was analyzed using GraphPad Prism version 10.4.0 (GraphPad Software, Boston, Massachusetts, USA).

## Results

### Cohort demographics

The study cohort consisted of 38 severely injured patients with an ISS of at least 18 (Table 1). The median age was 45.0 years, with an interquartile range (IQR) of 31.8 to 65.8 years. Of the total number of individuals in the study population (*n* = 38), nine were female (23.7%). A total of 23 severely injured patients (60.5%) sustained traumatic brain injuries (TBI). The median age of the healthy control population (*n* = 38) was 46.0 years (IQR 29.0–61.0), with 14 individuals being female (36.8%).

**Table 1:** Patient cohorts descriptive measures and available laboratory values at admission of the overall patient cohort as well as the healthy control cohort (unless otherwise specified values represent the median with interquartile range shown in brackets; P values between nominal variables assessed with Mann-Whitney test; P values between categorical variables assessed with Fisher´s exact test; ais = abbreviated injury scale; bp = blood pressure in mmhg ck = creatine kinase activity in U/l; gcs = glasgow coma scale; iss = injury severity score; ldh = lactate dehydrogenase activity in U/l; niss = new injury severity score; tbi = traumatic brain injury)

Admission Parameter	Whole Patient Cohort (*n* = 38) (Median + IQR)	Healthy Controls (*n* = 38) (Median + IQR)	*p* value of difference between groups
Age (years)	45 (31.8–65.8)	46 (29.0–61.0)	0.6763
N Female (%)	9 (23.7%)	14 (36.8%)	0.3180
N with TBI (%)	23 (60.5%)	-	
ISS	26 (22.0–34.3)	-	
NISS	34 (26.5–41.5)	-	
AIS Head	3.5 (3.0–5.0)	-	
AIS Face	2 (1.0–2.5)	-	
AIS Chest	3 (3.0–4.0)	-	
AIS Abdomen	2 (2.0–3.0)	-	
AIS Extremeties/Pelvis	3 (2.0–3.0)	-	
AIS External	1 (1.0–1.0)	-	
CK (U/L)	305 (213.5–769.5)	-	
LDH (U/L)	358 (261.5–536.0)	-	
Troponin T (ng/L)	11 (9.0–22.5)	-	
Base Excess	-1.2 (-3.2–1.1)	-	
Lactate (mmol/L)	1.7 (1.2–3.1)	-	
GCS	11 (3.8–15.0)	-	
Heartrate (bpm)	81.5 (73.8–99.3)	-	
Systolic BP (mmHg)	120 (100.0–140.0)	-	
Diastolic BP (mmHg)	75.5 (60.0–80.0)	-	
Shock Index	0.69 (0.57–0.99)	-	

Upon grouping according to the respective ALC dynamic (Table 2), 10 patients were assigned to the PL group and 10 to the RD group. The SR group comprised 12 severely injured patients. Furthermore, six patients were assigned to the NF group. The four groups demonstrated a statistically significant difference in age (*p* = 0.0274), with a median age of 74 years in the PL group (IQR 49.8–87.0), 46 years in the RD group (IQR 32.3–78.0), 36 years in the SR group (IQR 31.3–51.0) and 32.5 years in the NF group (IQR 24.5–48.3). The injury mechanisms were also displayed in (Suppl. Table 1). A comparison of the four groups revealed no statistically significant difference in injury severity, as indicated by the ISS, the New ISS (NISS), or the Abbreviated Injury Scale (AIS). Similarly, other parameters recorded at the time of admission, including creatine kinase (CK), lactate dehydrogenase (LDH), troponin T, base excess, lactate, Glasgow Coma Scale (GCS), heart rate, blood pressure (BP) and shock index, demonstrated no significant difference between all four groups.

**Table 2:** Patient cohorts depending on lymphocyte dynamics

Admission Parameter	Persistent lymphopenia *n* = 10 (Median + IQR)	Rapidly decreasing *n* = 10 (Median + IQR)	Slowly rising *n* = 12 (Median + IQR)	Normal fluctuations *n* = 6 (Median + IQR)	*p* value of difference between groups
Age (years)	74 (49.8–87.0)	46 (32.3–78.0)	36 (31.3–51.0)	32.5 (24.5–48.3)	0.0274
N Female (%)	2 (20.0%)	2 (20.0%)	2 (16.7%)	3 (50.0%)	0.5014
N with TBI (%)	5 (50.0%)	5 (50.0%)	10 (83.3%)	3 (50.0%)	0.2447
ISS	23.5 (18.8–29.3)	29 (24.0–41.0)	26.5 (22.0–39.5)	25.5 (23.5–34.3)	0.3109
NISS	31.5 (26.5–35.8)	41 (28.5–44.8)	30.5 (22.0–48.3)	34 (25.0–45.0)	0.6290
AIS Head	4 (2.0–4.0)	3 (2.8–5.0)	5 (3.0–5.0)	3.5 (1.5–4.8)	0.7602
AIS Face	1 (1.0–3.5)	2 (1.25–2.0)	1.5 (1.0–2.0)	2 (1.0–3.0)	0.9814
AIS Chest	3.5 (3.0–4.0)	4 (2.5–4.0)	3 (3.0–3.0)	3 (3.0–3.8)	0.4218
AIS Abdomen	2 (2.0–3.5)	2 (1.0–3.5)	2 (1.8–2.3)	3 (2.0–4.0)	0.7433
AIS Extremeties/Pelvis	3 (1.0–3.0)	3 (2.0–4.0)	3 (2.0–3.0)	3 (2.0–3.5)	0.6442
AIS External	1 (1.0–1.0)	-	1 (1.0–1.0)	1 (1.0–1.0)	-
CK (U/L)	236.4 (148.0–455.0)	391.5 (255.5–1043.0)	323 (170.3–730.3)	717 (253.5–1109.0)	0.2199
LDH (U/L)	338 (239.5–397.00)	379 (302.0–491.3)	323 (170.3–566.8)	438 (266.8–645.3)	0.5330
Troponin T (ng/L)	13 (9.8–47.3)	13 (8.5–38.3)	14 (8.5–20.5)	11 (6.0–19.0)	0.7981
Base Excess	-1.8 (-3.7– -0.5)	0.5 (-2.8–2.8)	-0.3 (-2.9–1.3)	-2.1 (-4.6–1.8)	0.4282
Lactate (mmol/L)	2.1 (1.2–3.4)	1.8 (1.2–3.3)	1.5 (1.2–2.1)	1.2 (1.0–4.0)	0.8466
GCS	12 (9.8–14.3)	9.5 (4.5–15.0)	11 (4.3–15.0)	3 (3.0–14.3)	0.4168
Heartrate (bpm)	90 (67.0–103.5)	78 (64.8–116.3)	81.5 (77.0–91.8)	84 (68.0–106.0)	0.9480
Systolic BP (mmHg)	127.5 (111.5–143.5)	125 (100.0–140.0)	119.5 (92.5–140.0)	100 (95.5–113.8)	0.2022
Diastolic BP (mmHg)	78 (69.5–85.0)	80 (60.0–85.0)	75.5 (60.0–80.0)	57 (51.3–78.0)	0.3780
Shock Index	0.71 (0.54–0.98)	0.61 (0.46–1.16)	0.71 (0.57–0.87)	0.85 (0.62–1.02)	0.7950

Descriptive measures and available laboratory values at admission of the favorable and unfavorable physical performative outcome groups (unless otherwise specified values represent the median with interquartile range shown in brackets; p values between categorical variables assessed using fisher’s exact test with Freeman-Halton extension; p values between continuous variables assessed with Kruskal-Wallis test; AIS = abbreviated injury scale; BP = Blood Pressure in mmHg; CK = creatine kinase activity in U/l; GCS = Glasgow Coma Scale; ISS = Injury Severity Score; LDH = Lactate Dehydrogenase activity in U/l; NISS = New Injury Severity Score; TBI = traumatic brain injury)

### Lymphopenia in severely injured patients

**Fig. 1:**
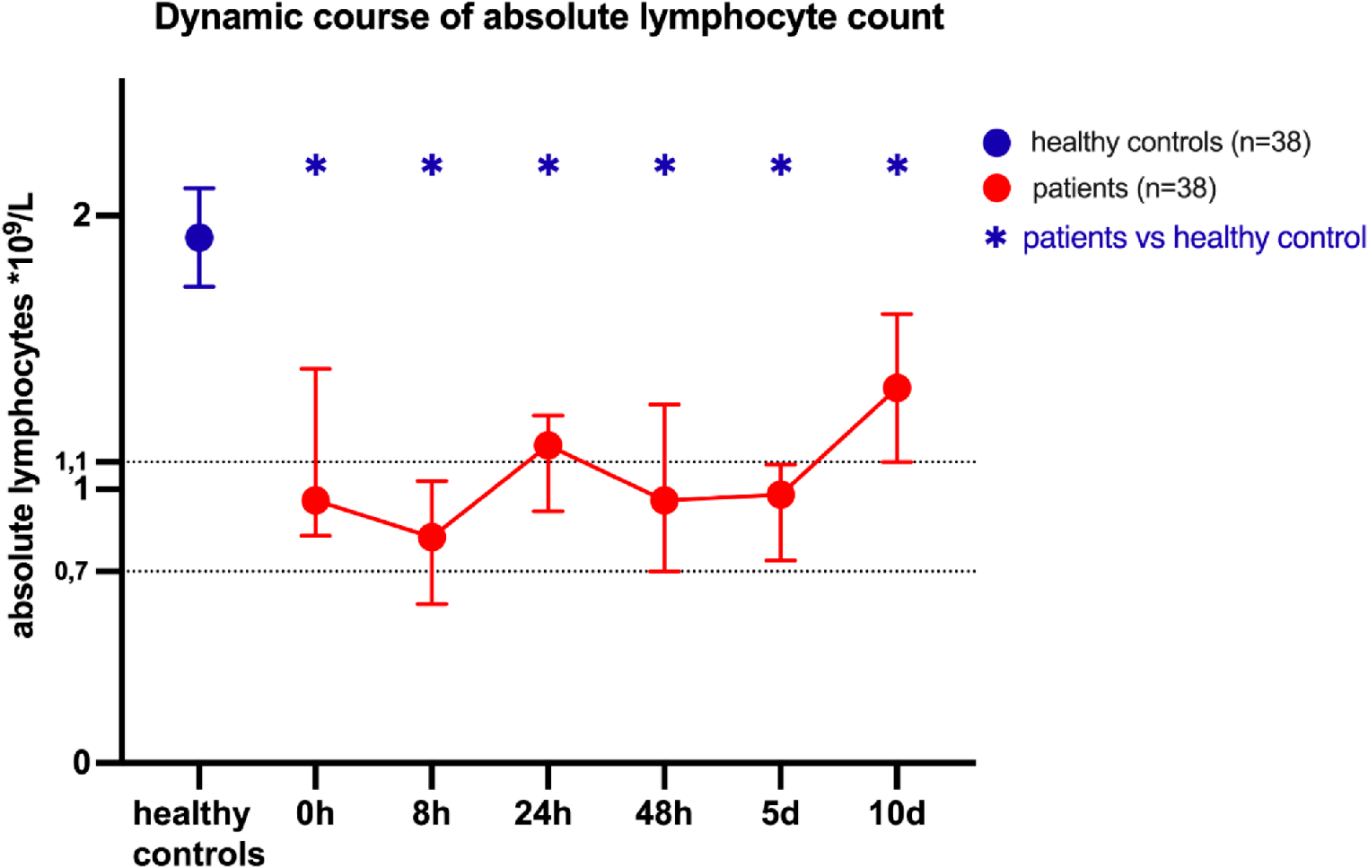
Dynamic course of absolute lymphocyte count in severely injured patients. Absolute lymphocyte count (ALC) measured in whole blood over a 10 day period in severely injured patients and in healthy volunteers on a single occasion. Median is presented with $\:\pm\:$95% confidence interval (CI). Lymphopenia was defined as an ALC $\:\le\:1.1*{10}^{9}$ lymphocytes per liter of whole blood. Severe Lymphopenia was defined as an ALC $\:\le\:0.7*{10}^{9}$ lymphocytes per liter of whole blood. Kruskal-Wallis test was employed to compare the statistical distribution of the data sets from the healthy volunteers and patients at each time point, assuming a nonparametric distribution. Threshold for significance *p* < 0.05

The severely injured patient cohort demonstrated a significantly lower ALC levels over a 10-day period in comparison to the healthy volunteers at all timepoints (Fig. 1). The median ALC in the severely injured patients remained within the range of lymphopenia, defined as an ALC $\:\le\:1.1\:\mathrm{x}\:10⁹\:$lymphocytes per liter of whole blood, until 8 h post-trauma and from 48 h to 5 days following the traumatic event. At the 24 h mark, the median ALC of the severe injury cohort exhibited a slight increase, reaching a median value >1.1 x$\:\:{10}^{9}$ lymphocytes per liter of whole blood. 10 days after the initial trauma, patients further partially recuperated their ALC, however, these values remained significantly lower than those observed in the healthy subjects throughout the whole observation period.

### Dynamic course of absolute lymphocyte count

**Fig. 2:**
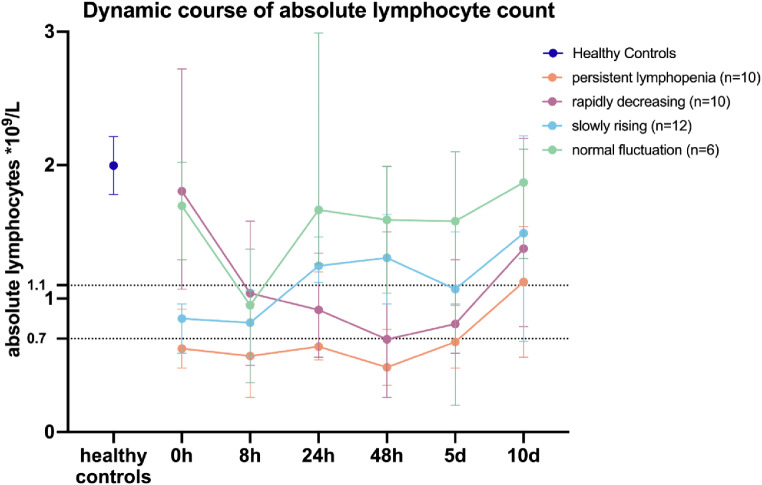
Grouping of severely injured patients into four dynamic patterns of absolute lymphocyte count. Absolute lymphocyte count (ALC) measured in whole blood over a 10 day period in severely injured patients and in healthy volunteers on a single occasion. Median is presented with $\:\pm\:$95% confidence interval (CI). Lymphopenia was defined as an ALC $\:\le\:1.1*{10}^{9}$ lymphocytes per liter of whole blood. Severe Lymphopenia was defined as an ALC $\:\le\:0.7*{10}^{9}$ lymphocytes per liter of whole blood. Group definitions whereby a 10% variance was accepted: persistent lymphopenia = severe lymphopenia for a minimum of 48 h; rapidly decreasing = initial normal ALC (ALC $\:>1.1*{10}^{9}$ lymphocytes per liter of whole blood), rapidly decreasing to lymphopenia latest at 48 h following the initial trauma, slowly rising = initial lymphopenia that decreases to normal ALC values (ALC $\:>1.1*{10}^{9}$ lymphocytes per liter of whole blood) latest at 48 h following the initial trauma, normal fluctuation = ALC values remaining within the normal range (ALC $\:>1.1*{10}^{9}$ lymphocytes per liter of whole blood) during the entire observation period

The entire severe injury cohort was subsequently classified according to their respective ALC dynamic pattern, which was observed throughout the duration of the study (Fig. 2). The four groups were designated as PL, RD, SR and NF over the 10-day observational period. The PL group displayed the lowest median ALC throughout the entirety of the study period, with severe lymphopenia persisting for a period of 5 days following the trauma. The SR group demonstrated lymphopenia for an 8-hour period, followed by a subsequent recovery to a normal ALC after 24 h. The RD group and the NF group initially exhibited an ALC that was within the normal range. During the observation period, the RD group exhibited a reduction in ALC to lymphopenia values over a period of 8 h until 5 days after the trauma. In contrast, the NF group demonstrated normal ALC throughout the entirety of the study, with the exception of the 8-hour time point, where the median ALC reached a value of $\:\le\:\:0.95\:\:\mathrm{x}\:10⁹\:$lymphocytes per liter of whole blood. 10 days following the initial trauma, all four groups demonstrated a recovery of their ALC to normal values, with a median ALC > 1.1 x$\:\:{10}^{9}$ lymphocytes per liter of whole blood. However, these values remained lower than the median ALC observed in the healthy control cohort.

Furthermore, to investigate these dynamics in an age-dependent manner, the patients were divided into two age groups (less than 60 years old and 60 years old or older) for separate analysis within each age group (Suppl. Figure 1). The four distinct dynamic courses of ALC observed in the younger patient cohort were found to be similar to those observed in the entire patient cohort (Suppl. Figure 1 A). In patients aged 60 years or older, no individual exhibited a SR ALC (Suppl. Figure 1B). Moreover, older severely injured patients displayed diminished lymphocyte levels across all dynamic subgroups when compared to their younger counterparts.

### Physical performative outcome

**Fig. 3:**
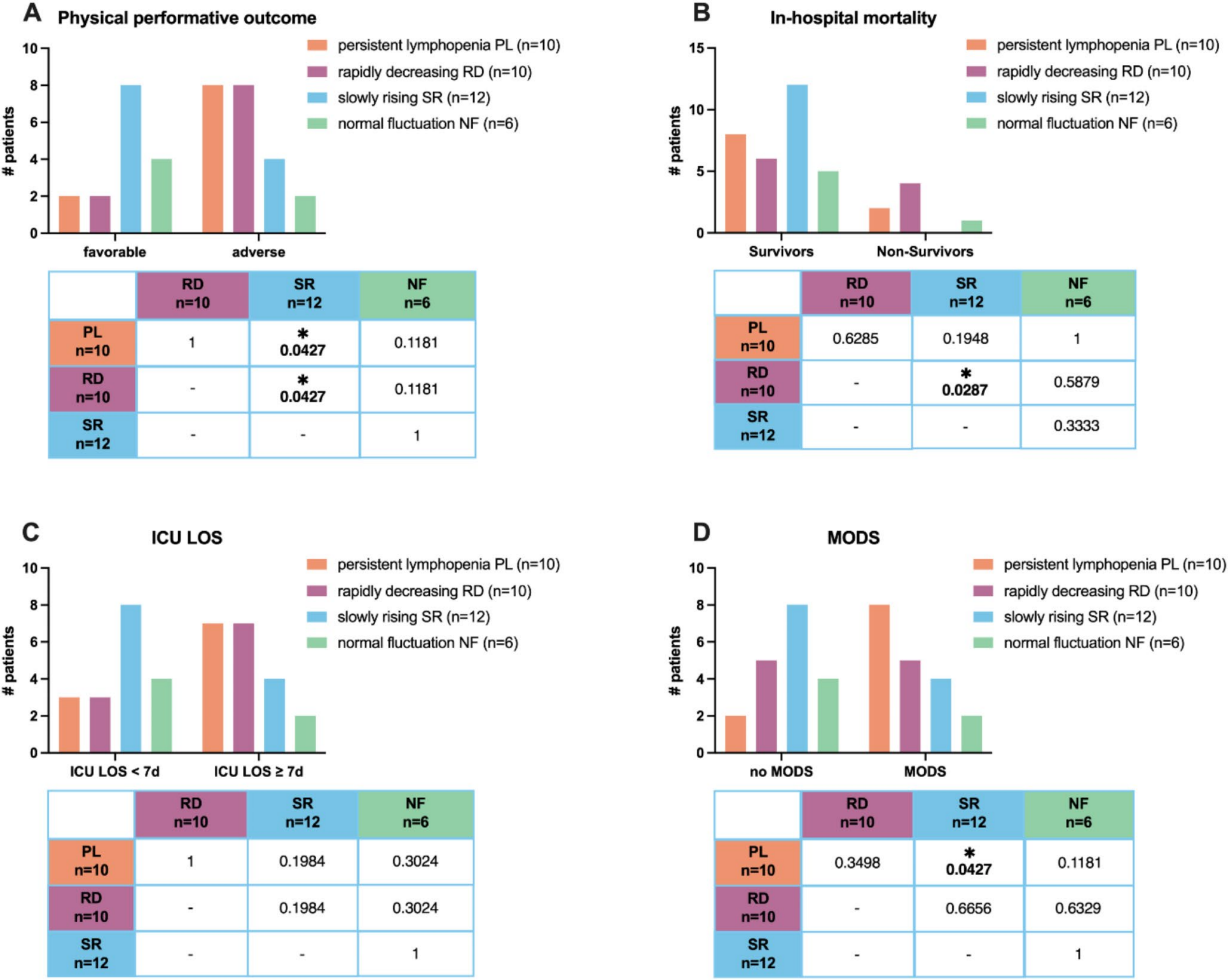
Comparison of clinical outcome depending on lymphocyte dynamic. Different outcomes within the four characteristics ALC groups. **A**) physical performative outcome: A favorable physical performative outcome was defined as patients who were discharged home either with or without ambulant nursing care. An adverse physical performative outcome was defined as discharge to a nursing home, acute or long-term care facility, a different hospital, or in-hospital death. **B**) in-hospital mortality. **C**) intensive care unit (ICU) length of stay (LOS) and **D**) multi-organ disfunction syndrome (MODS). MODS was defined as a sequential organ failure assessment (SOFA) score of six or greater on at least two consecutive days 48-hours post-admission. The upper graphs present the absolute numbers. The Fisher’s exact test was employed to assess the statistical difference between the groups. These results are presented in the tables. Threshold for significance *p* < 0.05

A favorable physical performative outcome was observed in 16 patients, whereas an adverse physical performative outcome was evident in 22 study patients (Fig. 3A).

The majority of patients with a favorable condition were in the SR group (50%; *n* = 8) (Fig. 3A). The remaining half of the patients with a favorable physical performative outcome were distributed among the NF group (25%; *n* = 4) and the PL (12.5%; *n* = 2) and RD (12.5%; *n* = 2) group. Consequently, the two groups with the fewest patients among the entire cohort who experienced a favorable physical performative outcome were PL and RD.

In contrast, most patients exhibiting an adverse physical performative outcome were classified as belonging to the PL (*n* = 8) and RD (*n* = 8) group (36.4% each). The SR group constituted only 18.2% (*n* = 4) of the total number of patients, while 9.1% of those demonstrating an adverse physical performative outcome showed NF of ALC (*n* = 2).

An examination of the physical performative outcome within each subgroup revealed that the majority of patients with PL (80.0%) and RD (80.0%) encountered adverse outcomes. In the SR and NF group a favorable physical performative outcome was observed in most patients (SR 66.7%; NF 66.7%).

A comparison of the distribution of favorable and adverse physical performative outcomes within each subgroup revealed a statistically significant difference (*p* = 0.0427) between the PL group and the SR group. Severely injured patients suffering from PL showed a statistically significant increase in adverse physical performative outcome compared to patients experiencing SR ALC, who demonstrated a higher prevalence of favorable physical performative outcome.

Similarly, a comparison between the RD group and the SR group revealed a statistically significant difference (*p* = 0.0427) between both groups too, with a greater prevalence of adverse outcomes observed in the RD cohort.

### In-hospital mortality

A total of 7 patients died during their hospital stay. Of the study patients who survived (*n* = 31), 38.7% were in the SR group (*n* = 12), 25.8% were in the PL group (*n* = 8), 19.4% were in the RD group (*n* = 6) and 16.1% were in the NF group (*n* = 5) (Fig. 3B).

Among non-survivors, 57.1% were classified as belonging to the RD group (*n* = 4). 28.6% of the subjects were part of the group experiencing PL (*n* = 2) and 14.3% showed NF in ALC (*n* = 1).

Among the entire study cohort, the SR group constituted most survivors, with no patients in this group dying. Most of the severely injured patients who died were part of the RD group. A comparative analysis of the in-hospital mortality between the RD and the SR group revealed a statistically significant difference (*p* = 0.0287).

### Intensive care length of stay

47.4% of all severely injured patients were discharged from the ICU within seven days (*n* = 18), while 52.6% remained in the ICU for a minimum of seven days (*n* = 20). The majority of patients who remained in the ICU for less than seven days (44.4%; *n* = 8) were part of the SR group (Fig. 3C). The residual three groups exhibited an almost similar proportion of patients who were in the ICU for less than seven days.

In contrast, most patients who required at least seven days of intensive care were classified as belonging to the PL (*n* = 7) and RD (*n* = 7) group (35% each). Conversely, only 20% were part of the SR (*n* = 4) and 10% in the NF group (*n* = 2).

A comparative analysis of the ICU LOS within the group experiencing PL and SR ALC revealed a trend (*p* = 0.1984) with a tendency for patients suffering from PL to require a longer ICU LOS. Similarly, a comparison of the RD group with the SR group indicated a trend towards a more prolonged ICU LOS in the RD group.

### Multi-organ dysfunction syndrome

The development of MODS was documented in 50% of the severe injury cohort (*n* = 19), while the other 50% of this cohort did not suffer MODS (*n* = 19). Among all patients who did not develop MODS, 42.1% were part of the SR group (*n* = 8) (Fig. 3D). The remaining patients were distributed among the residual three groups: 26.3% were part of the RD group (*n* = 5), 21.1% exhibited NF (*n* = 4) and 10.5% demonstrated PL (*n* = 2).

The predominance of severely injured patients who developed MODS were observed to be part of the PL group (42.1%; *n* = 8). In decreasing order of frequency, the RD (26.3%; *n* = 5), SR (21.1%; *n* = 4) and NF group (10.5%; *n* = 2) accounted for patients who developed MODS.

The largest proportion of patients who did not develop MODS exhibited a SR ALC, whereas most patients who developed MODS were part of the PL group. A comparison of the incidence of MODS between these two groups yielded a statistically significant difference (*p* = 0.0427) indicating that severely injured patients were more likely to develop MODS when additionally experiencing PL.

### Age-related impact on ALC dynamics

Patients were divided into two age groups (less than 60 years old and 60 years old or older) for separate analysis within each age group (Suppl. Figure 1,2). The four distinct dynamic courses of ALC observed in the younger patient cohort were similar to those observed in the overall patient cohort (Suppl. Figure 1 A). In patients aged 60 years or older, no individual exhibited SR ALC and in addition, older patients with severe injuries had decreased lymphocyte levels in all dynamic subgroups compared to their younger counterparts (Suppl. Figure 1B). Notably, no discernible difference in ALC was observed between the healthy subjects stratified by age.

Analogous to the ALC dynamics, the distribution of the outcome endpoints within the younger cohort is similar to the overall cohort (Suppl. Figure 2 A, C,E, G). The SR and NF groups relatively resembled the two largest groups correlated with favorable outcome, characterized by a favorable physical performative outcome (Suppl. Figure 2 A), increased survival rates (Suppl. Figure 2 C), a reduced ICU LOS (Suppl. Figure 2E) and a reduced incidence of MODS (Suppl. Figure 2G). Conversely, younger patients with RD ALC shared the highest proportion of adverse outcomes with the exception of MODS development. In the elderly cohort, patients tend to have adverse outcome endpoints regardless of the associated dynamic pattern, characterized by adverse physical performative outcome (Suppl. Figure 2B), higher mortality rates (Suppl. Figure 2D), longer ICU LOS (Suppl. Figure 2 F) and increased incidence of MODS (Suppl. Figure 2 H). However, the largest share of patients with adverse outcome were always from the PL group for all four outcome definitions. Examining the correlation of age and ALC, we found a non-significant trend of decreased ALC with age was observed in the severely injured patients (Suppl. Figure 3 A) as well as in the healthy volunteers (Suppl. Figure 3B).

## Discussion

Characteristic dynamics of ALC, clustered in four groups defined depending on their dynamic course, PL, RD, SR and NF, were established in a general ICU cohort of ~ 2k patients [11]. In this present study these four defined dynamic patterns correlate with clinical outcome in severely injured patients and correlate with clinical outcome (Fig. 3). Severely injured patients with either PL or a RD ALC appear to be prone to an adverse physical performative outcome (Fig. 3A), increased mortality rates (Fig. 3B), prolonged ICU LOS (Fig. 3C) and are at a higher risk of developing MODS (Fig. 3D). This is consistent with other studies that found the severity of lymphopenia associated with an increased risk of subsequent major infection or death [12]. In contrast, patients exhibiting NF or a SR ALC are more likely to have a favorable physical performative outcome (Fig. 3A), a shorter ICU LOS (Fig. 3C) and a decreased incidence of MODS (Fig. 3D). It should be noted that there is no difference in the in-hospital survival rates among severely injured patients with different ALC dynamics (Fig. 3B).

The monitoring of systemic ALC over time seems to be superior to singular measurements of ALC and may predict susceptibility of trauma patients to develop adverse clinic outcome such as adverse physical performative outcome, prolonged ICU LOS and the development of MODS. Although suggested by our findings, this should be elaborated in larger study cohorts and yet is consistent with the findings of Heffernan et al. who have demonstrated that persistent lymphopenia over the initial four days following a severe trauma was independently associated with a higher risk of mortality and with an increased hospital LOS [10].

Given the correlation between specific ALC dynamics and outcomes, the ALC in whole blood might serves as a valuable prognostic marker to define outcomes. Patients can be classified into a specific cohort at the latest at 48 h following the initial trauma. If future studies can prove the predictive behavior of dynamic ALC measurement this might facilitate clinical decision-making in the future. Lymphocyte counts are routinely obtained as part of the normal care of critically injured patients, the ALC is readily incorporated into clinical procedures and could represent a cost-effective option for developing personalized patient care for severely injured patients and a better planning of clinical resources such as intensive care capacity.

The exact mechanistic causes of lymphopenia in patients with severe trauma are not fully understood. Apoptosis appears to contribute to lymphopenia in severely injured patients [6, 13]. Previous studies indicate that lymphocytes in trauma patients demonstrate markedly elevated levels of apoptosis early after severe trauma, when compared to healthy individuals [3, 14]. The duration and extent of lymphopenia resulting from lymphocyte apoptosis are associated with an elevated risk of infection and mortality in patients with sepsis and other severe injuries such as trauma or burns [6]. However, the direct relationship between increased apoptosis-rates and adverse outcomes in trauma patients still needs to be proven.

Lymphopenia commences with an initial increase in T cell apoptosis, which is then followed by a period of T cell anergy [13]. Hence, apoptosis rates may return to normal once T cells become anergic [13], which may explain the PL or RD ALC observed in some groups (Fig. 2). Further research is needed to gain a better understanding of these mechanisms and their implications for different ALC dynamics.

A potential driver of apoptosis is the release of danger-associated molecular patterns (DAMPs) in response to injury [15]. DAMPs, like high mobility group box 1 protein (HMGB1), have the potential to initiate apoptosis [16]. The hypothesis that DAMPs induce apoptosis post trauma is corroborated by the observation that the median ALC levels of all four patient groups were consistently lower than those of the healthy controls throughout the observation period (Fig. 1). This observation is consistent with existing reports of trauma-related lymphopenia and immunosuppression [7, 8, 12].

A reduction in lymphocyte numbers is evident in all severely injured patients when compared to the healthy controls (Figs. 1 and 2). A non-significant reduction is observed even in cases where the patients do not meet the criteria for lymphopenia (Fig. 2). Similarly, a recovery is detectable in all severely injured patients after 10 days. Comparing our exclusive traumatic population versus the general ICU patient cohort of Pei et al. [11], we find an instant decrease of ALC after 8 h in the NF group (Fig. 2) which was not discovered in the study of Pei et al. as their observation timepoint included only admission and day one [11]. It is possible that trauma patients in our study could be considered as immune competent insofar as a potential lymphopenia-induced lymphoproliferation mechanism may instantly lead to a recovery of ALC. However, the ALC remained lower throughout the whole study period, and this remains speculative. Other studies indicate that the recovery trajectory of ALC is crucial for survival[10]. A rapid normalization within the initial post-trauma period is associated with improved outcomes [10]. Furthermore, delayed recovery can result in increased susceptibility to infection and higher mortality rates [10]. These findings are also supported by our results (Fig. 3).

It is unclear if a reduction in ALC in certain cases may be attributable to dilution effects resulting from bleeding, transfusion, or infusion following trauma. Interestingly Cao et al. demonstrated that the total volume administered to a patient within the first 24 h post trauma did not affect systemic ALC in these patients [17]. In our study cohort we did not find differences in the shock index on admission (Fig. 2), therefore we assume no bias for this cohort regarding dilution effects affecting ALC values on admission.

In accordance with the ALC classification by Pei et al. [11], we demonstrate in our study that severely injured patients can be classified into four distinct groups, each representing a unique ALC dynamic (Fig. 2). This suggests that the dynamic rather than mere presence of reduced ALC is influencing clinical recovery. Furthermore, the correlation between these ALC dynamics and specific outcomes highlights the importance of considering the dynamic nature of lymphopenia in severely injured patients. It is therefore of interest to examine differences between the patient cohorts depending on their ALC dynamic to evaluate whether the injury mechanism accounts for different dynamics or potentially medical preconditions.

Patients with underlying PL following a traumatic event are significantly older than those demonstrating NF (Table 2). No significant differences were identified between the two groups with respect to injury severity or other admission parameters. Immune cells undergo a process known as immunosenescence over a lifetime, which includes reduced systemic T cell counts in healthy elderly adults compared to younger subjects [18]. In the elderly severely injured patient population a higher share of patients revealed a PL or RD ALC as in the younger severely injured patient population, with no individuals in the ≥ 60 years group demonstrating a SR ALC (Suppl. Figure 1B). Consequently, the higher median age observed in the PL group could be explained by the fact that elderly patients present with lower ALC. Since the elderly cohort also showed a higher prevalence of adverse outcomes irrespective of dynamic patterns (Suppl. Figure 2B, D,F, H) they might be unable to recover from lymphopenia as effectively as younger patients, which might also account for the occurrence of PL in older patients.

Considering these observations, it is pertinent to inquire whether elderly severely injured patients are predisposed to adverse outcomes due to their advanced age and whether they are inherently unable to recuperate their ALC. The absence of a significant correlation between age and ALC levels in both patient and healthy cohorts (Suppl. Figure 3) challenges the notion of an age-dependent occurrence of certain outcomes.

In the RD group, the development of MODS could be attributed to the early decrease of ALC levels which itself might be attributed to early lymphocyte activation. This hypothesis is supported by Manson et al.., who found that early changes in lymphocyte populations, including activation within 2 h of trauma, and lymphopenia 48 h post-trauma were associated with the development of MODS. Patients who developed lymphopenia 48 h post-trauma had activated lymphocytes on admission [8]. The ALC dynamic in the RD group shows a tendency of higher incidence of MODS although not statistically significant (Fig. 3D). We found the RD group trended to be more frequently represented in patients suffering from adverse physical performative outcome, MODS, ICU LOS of more than 7 days and intra-hospital mortality in patients older than 60 years (Suppl. Figure 2B, D,F, H) and younger (Suppl. Figure 2 A, C,E, G). Our results and the findings by Mason et al. [8] indicate that in order to prevent MODS development functional and quantitatively adequate numbers of lymphocytes are necessary. It is not well understood how lymphocytes contribute to the prevention of MODS development immunologically. However, our data provides a rationale for future studies for when an intervention likely has to happen to benefit the patient, which would be within 8 h post trauma before the ALC drops below normal levels.

The majority of severely injured patients in this study exhibited TBI (Table 1), which has been demonstrated to have an immune modulatory effect [19]. TBI has the capacity to induce changes in both humoral and cellular immunity, including reduced relative CD3 + lymphocyte counts in the early post-trauma period[20]. This finding potentially provides a biological rationale for the reduced ALC observed in most severely injured patients early after injury. However, while a significant proportion of patients across all four groups exhibited TBI (Table 2), a divergent dynamic was identified, with the SR and NF groups demonstrating the capacity to enhance their ALC. Further research is necessary to comprehensively elucidate the intricate relationship between TBI, severe injuries and ALC.

### Study limitations

It should be noted that the limited sample size and heterogeneity of the traumatic events could have influenced the results. Due to the limited sample size, it was statistically not feasible to conduct a subgroup analysis of patients presenting with or without TBI, which is known to have an immune modulatory effect on its own [19]. Potential sampling bias cannot be ruled out given the limited patient numbers. All patients in our study population sustained multiple injuries; however, not all met the Newcastle definition of polytrauma [21]. A comparison of our study population with the 3-year average of all polytraumatized patients in Germany, depicted in the annual report (2023) from the German Society for Trauma Surgery (DGU)[22] revealed that our limited study population is comparable to the mentioned 3-year average with the exception of the ISS (Average (mean) study population: age (years) 49, female patients (%) 23.7, ISS 28.4; 3-year average (mean) national report 2023: age (years) 53.2, female patients (%) 30, ISS 20.0). As the national report does not provide individual data, a statistical comparison to our population could not be displayed here. We believe primary distinction in terms of ISS can be attributed to the exclusion of trauma patients with an ISS of less than 18. Moreover, a statistical differentiation between blunt and penetrating trauma could not be conducted to limited patient numbers suffering from penetrating trauma. Furthermore, it cannot be entirely excluded that some of the observed effects may be age-dependent (Suppl. Figures 1 and 2). Further investigation is recommended, particularly to determine whether ALC levels could serve as biomarkers in younger and older individuals. This study did not evaluate the functional activation of the cells. It is possible that the observed cells are not functionally active and would have to be addressed in future studies.

## Conclusion

Our findings indicate that the dynamics of lymphocyte levels over 48 h may designate clinical outcome more precisely than the admission lymphopenia alone. With this study representing a feasibility study, the dynamic course of ALC may serve as a practical approach in clinical decision-making and could serve as an indicative biomarker for the incidence of potential outcomes in severely injured patients when confirmed in additional studies. We believe this provides a rationale to validate our findings in a larger patient cohort. PL and RD ALC are associated with an elevated risk of an adverse physical performative outcome, prolonged ICU LOS, and an increased incidence of MODS. Severely injured patients exhibiting NF or SR ALCs tend to have a more favorable outcome.

Taken together, the ALC dynamic may help identify severely injured patients at risk at an early stage, thus potentially supporting more informed clinical decision-making and enhanced patient care.

## Electronic supplementary material

Below is the link to the electronic supplementary material.

Supplementary Material 1

Supplementary Material 2

## Data Availability

The data are available from the corresponding author upon reasonable request.
