# Supplementary material for: Absolute lymphocyte count trajectory predicts clinical outcome in severely injured patients
Source: Eur J Trauma Emerg Surg. 2025 May 2;51(1):190. doi: 10.1007/s00068-025-02864-0 (PMC12048453; doi:10.1007/s00068-025-02864-0)
Supplement: Supplementary file 2 — Supplementary Material 2 [file 68_2025_2864_MOESM2_ESM.docx]

# **Supplemental Material**

| **Accident Mechanism** | | **Persistent lymphopenia**  **n=10**  **(n)** | **Rapidly decreasing**  **n=10**  **(n)** | **Slowly rising**  **n=12**  **(n)** | **Normal fluctuations**  **n=6**  **(n)** |
| --- | --- | --- | --- | --- | --- |
| Blunt Trauma | | **10** | **10** | **11** | **6** |
|  | MVCs | 1 | 5 | 7 | 5 |
|  | Pedestrian-Vehicle Collisions | 2 | 1 | 1 | - |
|  | Falls | 2 | 1 | - | 1 |
|  | Falls from Heights | 3 | 3 | 3 | - |
|  | Assaults | 2 | - | - | - |
| Penetrating Trauma | | **-** | **-** | **1** | **-** |

### **Supplemental Table 1: Accident Mechanisms**

The number of patients with respective accident mechanisms were grouped into blunt and penetrating trauma. Further subdivision of blunt trauma into MVCs (= Motor-Vehicle-Collisions; rapid deceleration injuries, seatbelt injuries, airbag deployment injuries), Pedestrian-Vehicle-Collisions, Falls (= high energy falls, ground-level falls), Falls from Heights (= falls from scaffolding, ladders or construction-related falls) and Assaults (= punches, kicks, blunt objective strikes).


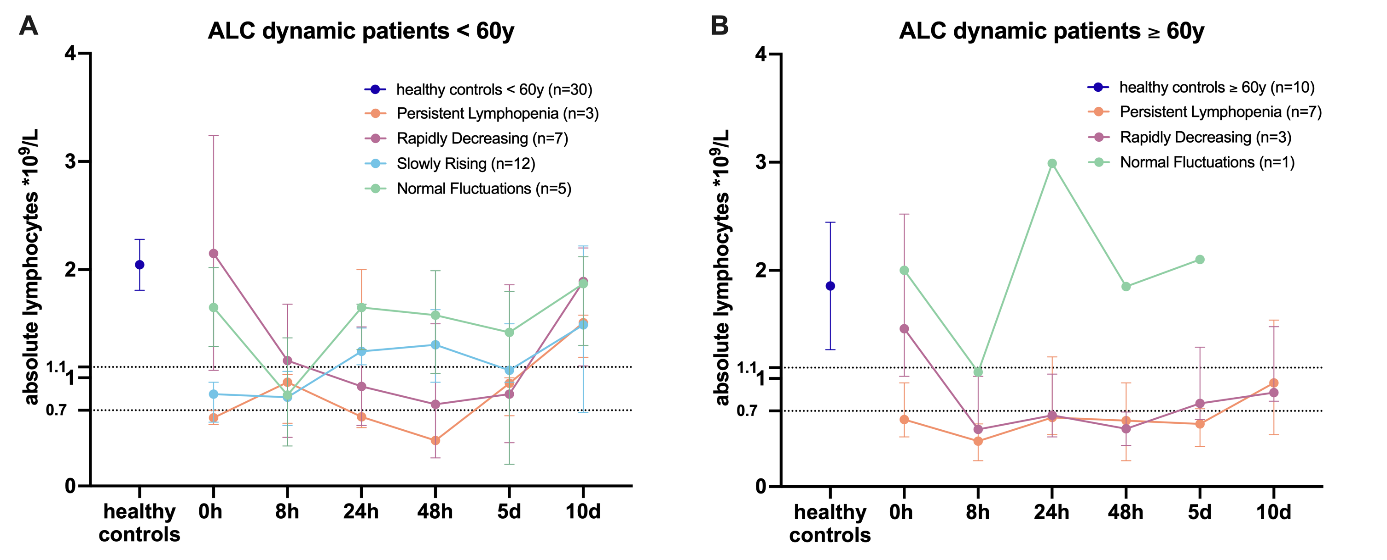


### **Supplemental Figure 1: Dynamic course of Absolute Lymphocyte Count in young and elderly individuals**

Absolute lymphocyte count (ALC) measured in whole blood over a ten-day period in A) severely injured patients and healthy controls younger than 60 years and B) severely injured patients and healthy volunteers 60 years old or older. Median is presented with $\pm$95% confidence interval (CI). Lymphopenia was defined as an ALC $\leq1.1*{10}^{9}$ lymphocytes per liter of whole blood. Severe Lymphopenia was defined as an ALC $\leq0.7*{10}^{9}$ lymphocytes per liter of whole blood. Group definitions whereby a 10% variance was accepted: persistent lymphopenia = severe lymphopenia for a minimum of 48 hours; rapidly decreasing = initial normal ALC (ALC $>1.1*{10}^{9}$ lymphocytes per liter of whole blood), rapidly decreasing to lymphopenia latest at 48 hours following the initial trauma, slowly rising = initial lymphopenia that decreases to normal ALC values (ALC $>1.1*{10}^{9}$ lymphocytes per liter of whole blood) latest at 48 hours following the initial trauma, normal fluctuation = ALC values remaining within the normal range (ALC $>1.1*{10}^{9}$ lymphocytes per liter of whole blood) during the entire observation period.


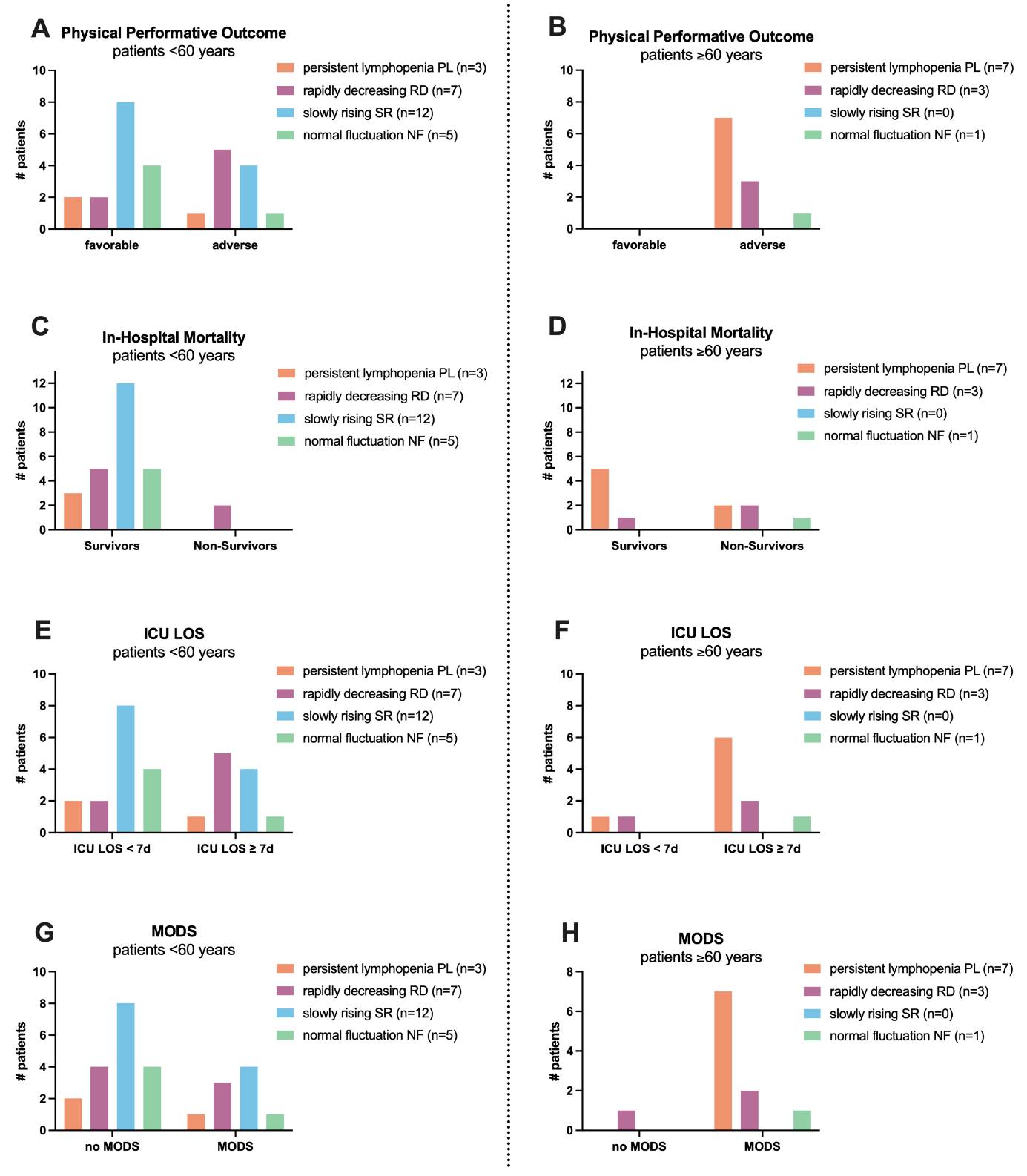


### **Supplemental Figure 2: Different outcome endpoints within each ALC subgroup in young and elderly individuals**

Patients were divided by age into younger (< 60 years old) and older ($\geq60$years old). Different outcomes within the four characteristics ALC groups are demonstrated in an age-comparison manner, including A,B) physical performative outcome at discharge, C,D) in-hospital mortality, E,F) intensive care unit (ICU) length of stay (LOS) and G,H) multi-organ disfunction syndrome (MODS). The upper graphs present the absolute numbers. The Fisher’s exact test was employed to assess the statistical difference between the groups. These results are presented in the tables. Threshold for significance p<0.05


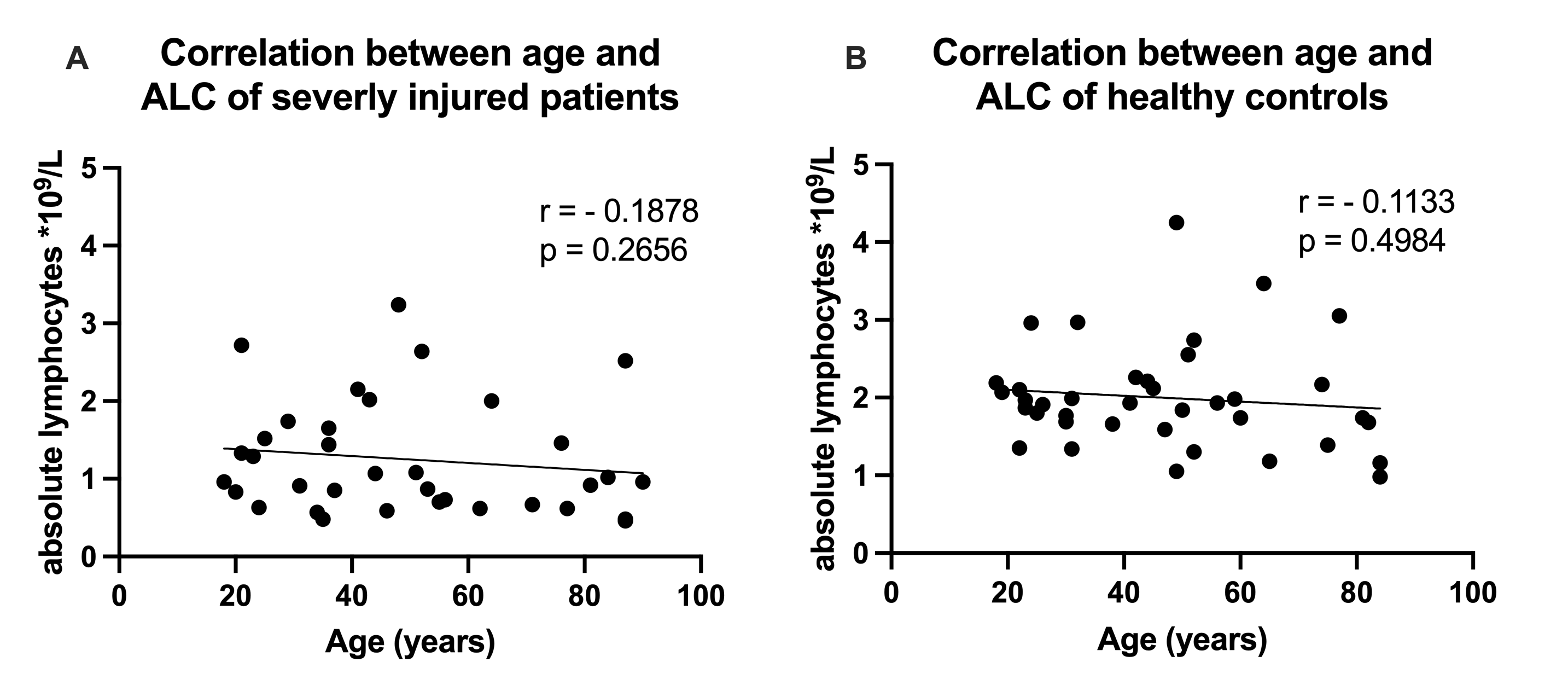


### **Supplemental Figure 3: Correlation of age and absolute lymphocyte count**

Correlation between age and absolute lymphocyte count (ALC) in whole blood of A) severely injured patients at the 0-hour time point and B) healthy individuals. Results of Spearman-Correlation test assuming a nonparametric distribution. Threshold for significance p < 0.05
